# Supplementary material for: Topological states in multi-orbital HgTe honeycomb lattices
Source: Nat Commun. 2015 Mar 10;6:6316. doi: 10.1038/ncomms7316 (PMC4366513; doi:10.1038/ncomms7316)
Supplement: Supplementary Information — Supplementary Figures 1-5, Supplementary Tables 1-2, Supplementary Notes 1-3, and Supplementary References [file ncomms7316-s1.pdf]

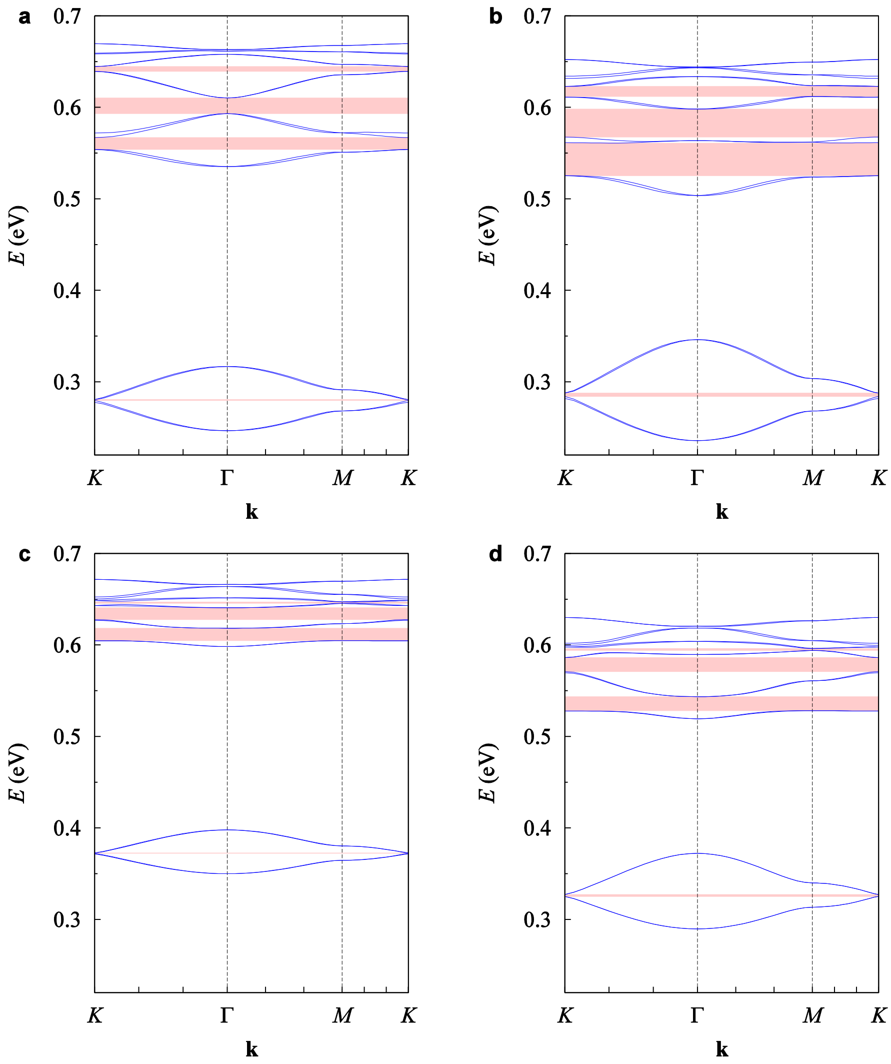

Supplementary Figure 1: Conduction bands in lattices of HgTe nanocrystals. Band dispersion resulting from the atomistic tight-binding calculations. Topologically non-trivial gaps are indicated by pink shaded regions. Each nanocrystal has a truncated nanocube shape. Truncation factor: q=0.25 (**a**,**c**) or q=0.45 (**b**,**d**). Honeycomb lattice spacing: a=5.9 nm (**a**,**b**) or a=6.8 nm (**c**,**d**).

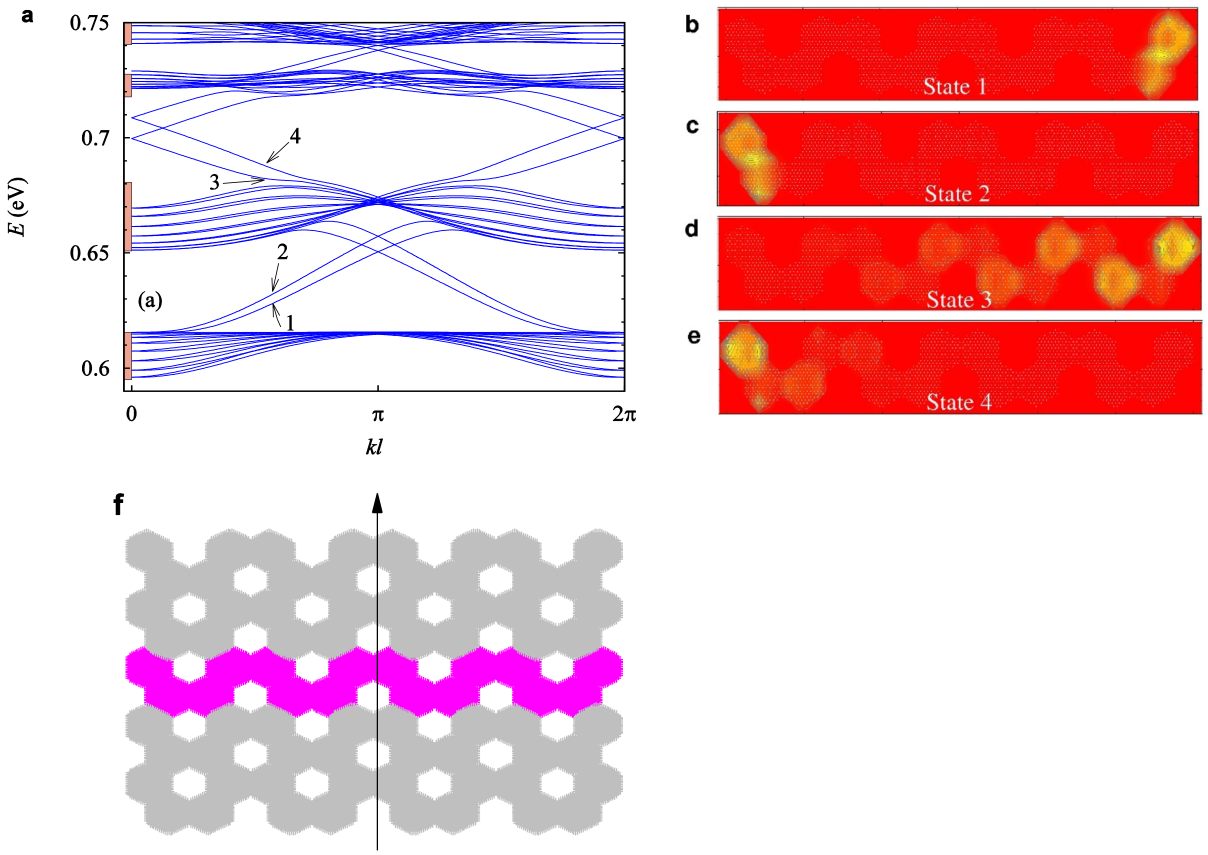

Supplementary Figure 2: Ribbon with broken inversion symmetry. Atomistic tight-binding calculations for a honeycomb ribbon of HgTe nanocrystals, with broken inversion symmetry. The nanocrystals have a truncated nanocube shape (truncation factor q=0.5, honeycomb lattice spacing a=5.0 nm). A single plane of atoms is removed from the right side of the ribbon in order to break the inversion symmetry (not shown). **f**, schematic view of the ribbon. The unit cell of 16 nanocrystals, shown in magenta, is reproduced periodically along the direction indicated by the arrow. **a**, dispersion of the p bands. The position of the bulk bands is indicated by pink vertical bars along the left axis. **b,c,d,e**, 2D plots of the wave functions of four states calculated at $k = 0.3\times 2\pi/l$ where l is the length of the unit cell. The labels 1 (**b**), 2 (**c**), 3 (**d**) and 4 (**e**) refer to the states indicated in **a**. The plots are restricted to the unit cell of the ribbon. The white dots indicate the atoms.

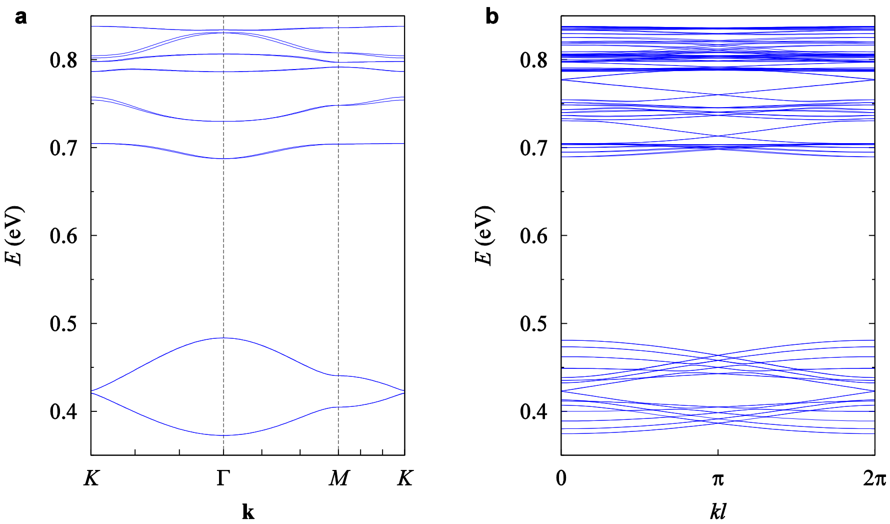

Supplementary Figure 3: Band structure for lattices of spherical nanocrystals. Conduction band dispersion resulting from the atomistic tight-binding calculation for honeycomb superlattices of spheres (diameter D=5 nm) connected by cylinders (diameter d=0.4 D). **a**: bulk (gap between the lowest p bands = 25 meV). **b**: armchair nanoribbon composed of sixteen nanocrystals per unit cell.

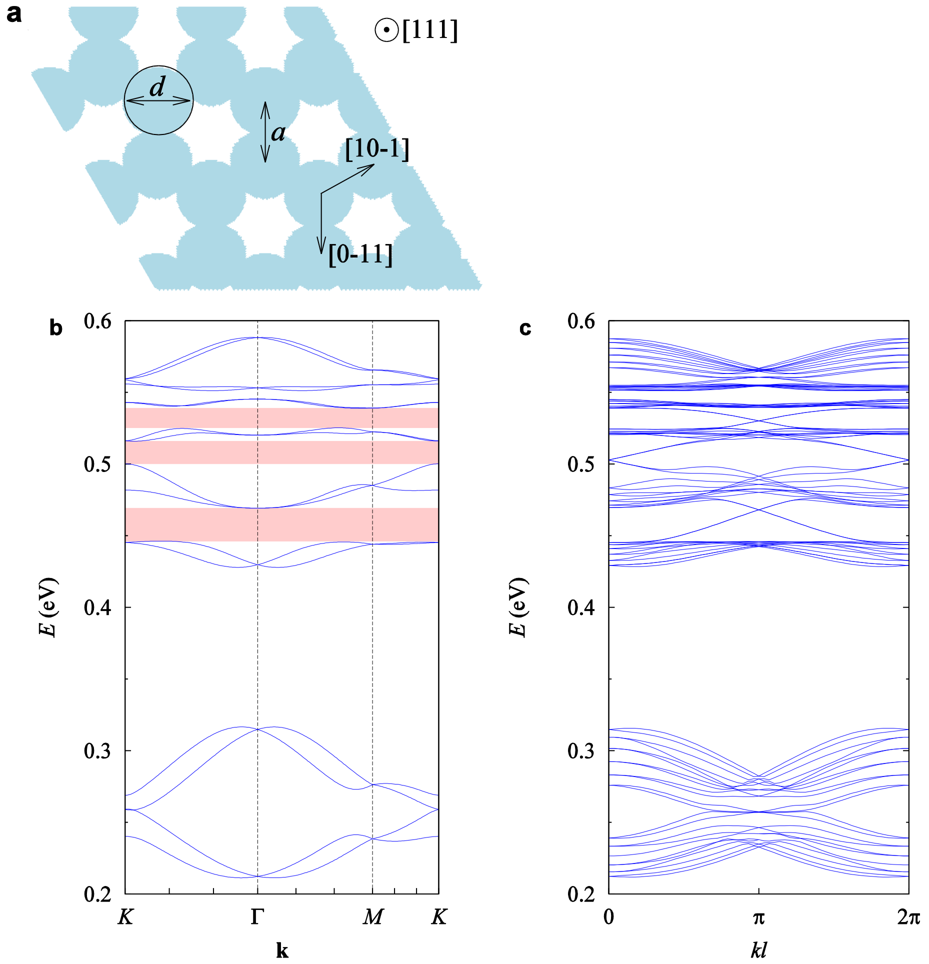

Supplementary Figure 4: Band structures for lattices of cylinders. **a**, top view of an assembly of vertical cylinders forming a honeycomb lattice of HgTe (cylinder diameter d = 1.02a = 7.0 nm, layer thickness t = 5.3 nm, a is the lattice spacing). Crystallographic axes of HgTe are indicated. **b**, corresponding conduction band dispersion resulting from the atomistic tight-binding calculation. Non-trivial gaps are indicated by pink shaded regions. **c**, band structure of a zigzag ribbon formed of 12 cylinders per unit cell.

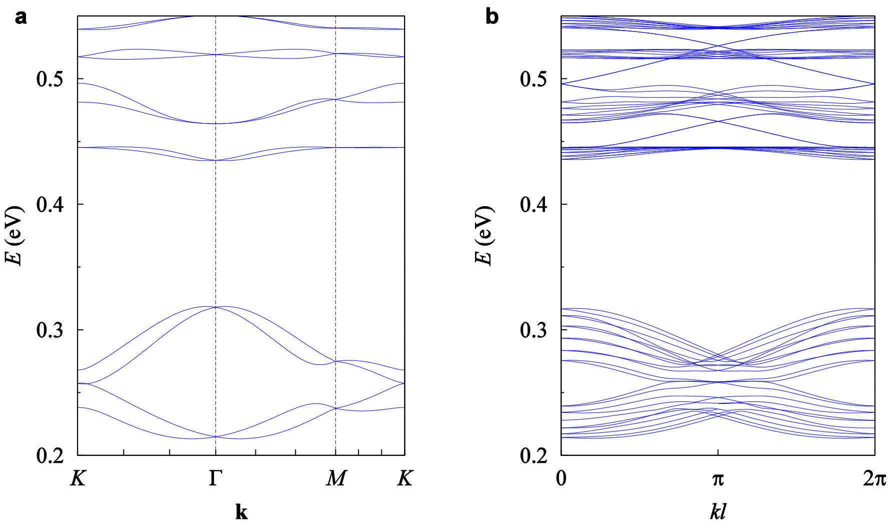

Supplementary Figure 5: Effective model for a lattice of cylinders. a, conduction band dispersion calculated using the model Hamiltonian. **b**, band structure of a zigzag ribbon formed by 12 sites per unit cell.

	a = 5.0 nm	a = 5.9 nm	a = 6.8 nm
q = 0.25	17.2	13.1	13.7
q = 0.30	17.2	13.6	13.7
q = 0.35	15.3	13.6	32.0
q = 0.40	15.3	36.2	32.0
q = 0.45	35.4	36.2	15.7
q = 0.50	35.4	18.6	15.7

Supplementary Table 1: Energy gap in the *p* bands. Energy gap (meV) between the lowest p bands in honeycomb lattices of HgTe nanocrystals for different values of the truncation factor q and the honeycomb lattice spacing a. The same gap predicted for two successive values of q means that the honeycomb lattices are identical, the number of atoms per nanocrystal varying discontinuously with q.

on-site	NN hopping	Rashba SOC	intrinsic SOC
$E_s = 266$	$V_{ss\sigma} = -17.2$	$\gamma_{ss\sigma} = 5.11$	$\lambda^s_{\mathrm{ISO}} = 0.3$
$E_{p_x} = 493$	$V_{pp\sigma} = 28.9$	$\gamma_{pp\sigma} = 4.77$	$\lambda^p_{\mathrm{ISO}} = 14.1$
$E_{p_y} = 493$	$V_{pp\pi} = -0.6$	$\gamma_{pp\pi} = 0.00$	
$E_{p_z} = 698$	$V_{sp\sigma} = 24.2$		

Supplementary Table 2: Parameters of the effective model. Parameters (meV) used for the model for the HgTe superlattice described in Supplementary Fig. 5a. E_s, E_{p_x}, E_{p_y}, and E_{p_z} are the onsite energies on the s, p_x, p_y, and p_z orbitals, respectively. $V_{ss\sigma}$, $V_{pp\sigma}$, $V_{pp\pi}$, and $V_{sp\sigma}$ are the hopping parameters, following the notations of Ref. [1]. $\gamma_{ss\sigma}$, $\gamma_{pp\sigma}$, and $\gamma_{pp\pi}$ are the terms describing the Rashba SOC, following the same notations. The intrinsic SOC is defined by λ^s_{ISO} and λ^p_{ISO} on s and p orbitals, respectively.

Supplementary Note 1
Influence of size on lattices of HgTe nanocrystals.

When we vary the lattice spacing a (3—8 nm) and the nanocube truncation (q between 0.25 and 0.5) in lattices of HgTe nanocrystals, all band structures have similar behaviour. Examples of band structure are shown in Supplementary Fig. 1. The presence of well-separated s and p bands with helical gaps is quite general, even if the width of the gaps may vary substantially depending on the geometry of the superlattices. The overall shape of the s bands is always the same, whereas for the p bands the variations are more important because not only the nearest-neighbour hopping, but also the respective positions of the p_x , p_y and p_z states depend on nanocrystal size and truncation. However, the lowest p band is always detached from the next higher one. The width of the lowest gap in the p bands is given in Supplementary Table 1 for different values of a and q.

Supplementary Note 2
Edge localization in ribbons of HgTe nanocrystal superlattices

In this section, we present additional results on ribbons made from lattices of HgTe nanocrystals. We consider the same nanocrystals as in Figs. 1 and 2 (q=0.5, a=5.0 nm) but we investigate a ribbon in which the two edges are not symmetric by inversion. First, we discuss the effect of this asymmetry on the band structure. Second, we present plots of the wave functions.

When the two edges of the ribbon are equivalent by inversion symmetry, the topological edge states on the opposite sides of the ribbon are quasi-degenerate for each value of k, the coupling between opposite edge states being negligible (Fig. 2). In order to observe the effect of geometry on the results, we have also considered a ribbon in which the inversion symmetry has been broken. For that purpose, we have removed all atoms of the last atomic plane on the right side of the ribbon and we have saturated the broken bonds with pseudo-hydrogen atoms. Supplementary Fig. 2a shows that the edge states are preserved thanks to their topological

protection but their degeneracy at a given k is lifted due to the asymmetry between the two sides of the ribbon.

The 2D plots of the wavefunctions of the four states denoted 1−4 in Supplementary Fig. 2a for the asymmetric ribbon are shown in Supplementary Figs. 2b−e. These states calculated at $k = 0.3\times2\pi/l$ are strongly localized on the edges of the ribbon. State 3 is more delocalized than the other three states because it lies very close to the bulk band edge.

Supplementary Note 3
Band structures of honeycomb lattices of vertical cylinders

We have investigated a third type of honeycomb structure composed of HgTe cylinders. The axes of the cylinders are parallel to each other and are organized on a honeycomb lattice (Supplementary Fig. 4a). Such structures could be fabricated from a HgTe layer, grown for example by gas-phase approaches. The honeycomb nanogeometry is then defined using nanoscale lithography.

Quite similar band structures are obtained for these lattices (Supplementary Fig. 4b). Once again, s and p-like bands can be easily identified. The Rashba SOC is in general much larger than for lattices of nanocrystals due to a stronger coupling between neighbouring sites. Interestingly, a very similar behaviour was found for superlattices of spheres connected by cylinders when the coupling between neighbouring spheres is strong, i.e., for large values of d/D (Fig. 3). As a consequence of the large Rashba SOC, the gap in the s sector is closed. Non-trivial gaps remain in the p bands, for many configurations that we have investigated, in spite of larger spin splitting. However, the larger Rashba SOC tends to increase the dispersion of the lowest p band.

Once again, the results of the atomistic TB calculations are well described by the effective model (Supplementary Table 2 and Supplementary Fig. 5a). Only the highest bands of Supplementary Fig. 4b are not reproduced by the effective model because they involve higher-energy orbitals which are not considered in the model. The topological properties of the bands are demonstrated by edge-state analyses in ribbons, using the atomistic TB calculations (Supplementary Fig. 4c) or the effective TB model (Supplementary Fig. 5b) which give results in excellent agreement. The non-trivial topology of the bands is confirmed by calculations of the Z_2 topological invariants using the effective-model Hamiltonian.

Supplementary Reference

[1] Slater, J. C. & Koster, G. F. Simplified LCAO method for the periodic potential problem. *Phys. Rev.* **94**, 1498–1524 (1954).
